# Theoretical Photoelectron Spectroscopy of Quadruple-Bonded Dimolybdenum(II,II) and Ditungsten(II,II) Paddlewheel Complexes: Performance of Common Density Functional Theory Methods

**DOI:** 10.1021/acsomega.4c00269

**Published:** 2024-03-04

**Authors:** Abhik Ghosh, Jeanet Conradie

**Affiliations:** † Department of Chemistry, UiT − the Arctic University of Norway, N-9037 Tromsø, Norway; ‡ Department of Chemistry, University of the Free State, P.O. Box 339, Bloemfontein 9300, Republic of South Africa

## Abstract

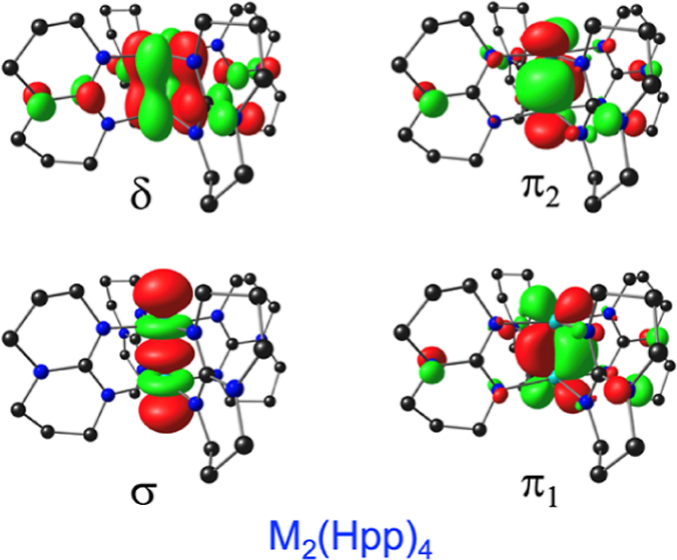

We have revisited the gas-phase photoelectron spectra of quadruple-bonded dimolybdenum(II,II) and ditungsten(II,II) paddlewheel complexes with modern density functional theory methods and obtained valuable calibration of four well-known exchange–correlation functionals, namely, BP86, OLYP, B3LYP*, and B3LYP. All four functionals were found to perform comparably, with discrepancies between calculated and experimental ionization potentials ranging from <0.1 to ∼0.5 eV, with the lowest errors observed for the classic pure functional BP86. All four functionals were found to reproduce *differences* in ionization potentials (IPs) between analogous Mo_2_ and W_2_ complexes, as well as large, experimentally observed ligand field effects on the IPs, with near-quantitative accuracy. The calculations help us interpret a number of differences between analogous Mo_2_ and W_2_ complexes through the lens of relativistic effects. Thus, relativity results in not only significantly lower IPs for the W_2_ complexes but also smaller HOMO–LUMO gaps and different triplet states relative to their Mo_2_ counterparts.

## Introduction

**Scheme 1:**
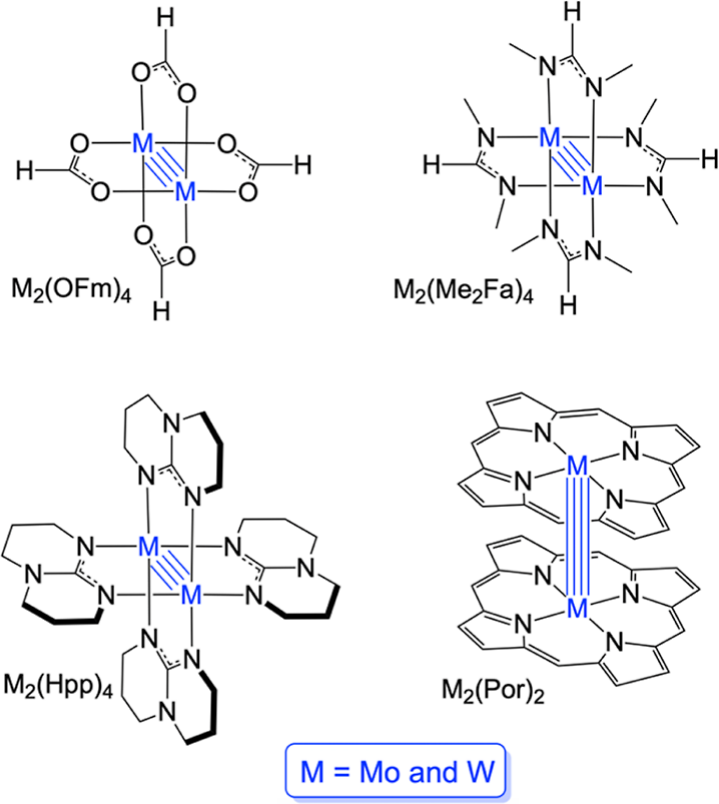
Quadruple-Bonded Compounds Studied in This Work

Conceptualized by Cotton nearly 60 years ago,^1−3^ metal–metal quadruple bonds are an icon of inorganic chemistry.^4^ They vary remarkably in terms of their electronic properties such as ionization potentials (IPs), electron affinities, redox potentials, the nature of the frontier orbitals, HOMO–LUMO gaps, and singlet–triplet gaps.^4−6^ The critical gas-phase photoelectron spectroscopy (PES) measurements,^7−14^ however, were made largely in the latter half of the last century and still remain inadequately explored with modern density functional theory (DFT) methods.^13−15^ We recently made an effort to close this knowledge gap with a comparative DFT study of quadruple-bonded metalloporphyrin^16^ and metallocorrole^17^ dimers.^18^ Here, we have extended these studies to *nonporphyrinoid* dimolybdenum(II,II) and ditungsten(II,II) paddlewheel complexes. We have examined three series of compounds—M_2_(OFm)_4_, M_2_(Me_2_Fa)_4_, and M_2_(Hpp)_4_—and compared the results with those for M_2_(Por)_2_, where OFm = formate, Me_2_Fa = *N*,*N*′-dimethylformamidinate, Hpp = hexahydropyrimidinopyrimidine, Por = unsubstituted porphyrin dianion, and M = Mo and W (Scheme 1). The results afford not only valuable calibration of the performance of common exchange–correlation functionals but also insights into periodic trends and relativistic effects as they pertain to metal–metal quadruple bonds. For transition metals, the two key scalar relativistic effects (as distinguished from spin–orbit coupling effects) are a stabilization of s orbitals and a destabilization of d orbitals. For a broader introduction to the subject, the reader may consult a nontechnical review article by Pyykkö^19^ and a popular science account in *American Scientist* by one of us.^20^ This study adds to our growing appreciation of relativistic effects in coordination chemistry.^21−25^

## Results and Discussion

**Figure 1:**
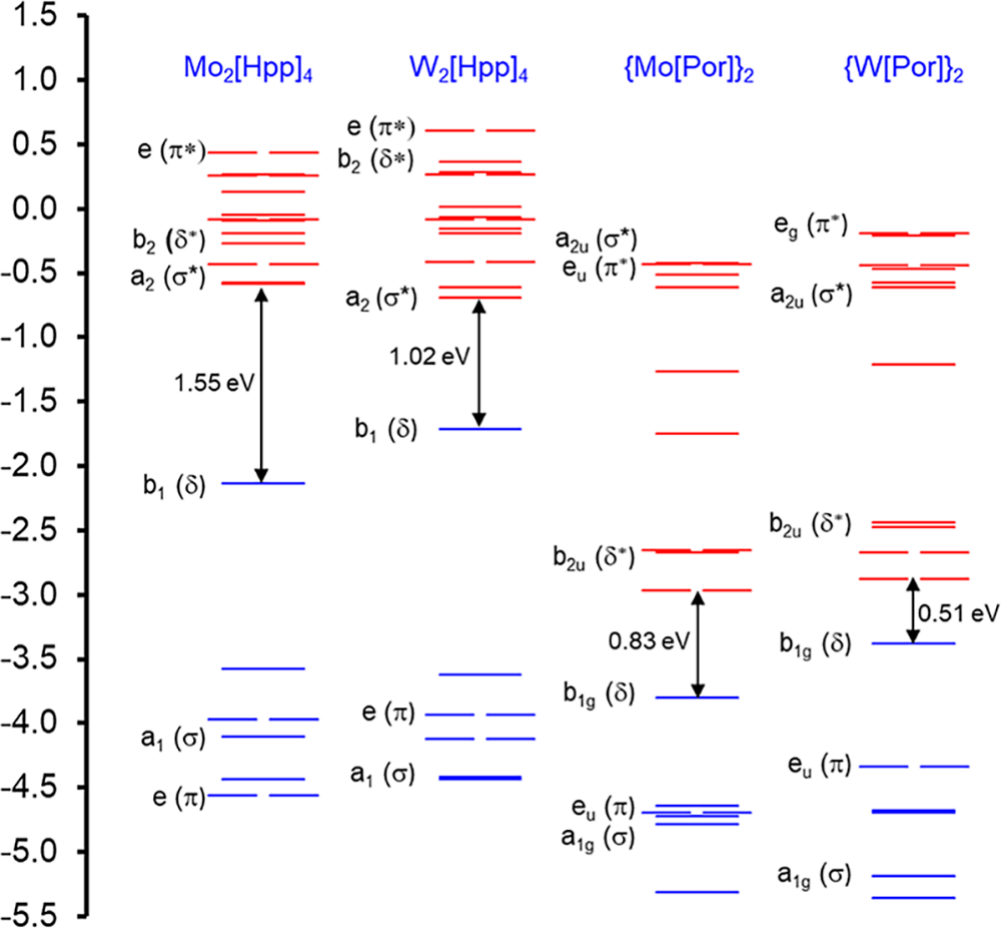
Comparative OLYP-D3/ZORA-STO-TZ2P MO energy level diagram (eV) for M_2_(Hpp)_2_ (*D*_4_) and M_2_(Por)_2_ (*D*_4*h*_), where M = Mo and W. Also indicated are MO irreps for the point group in question.

**Figure 2:**
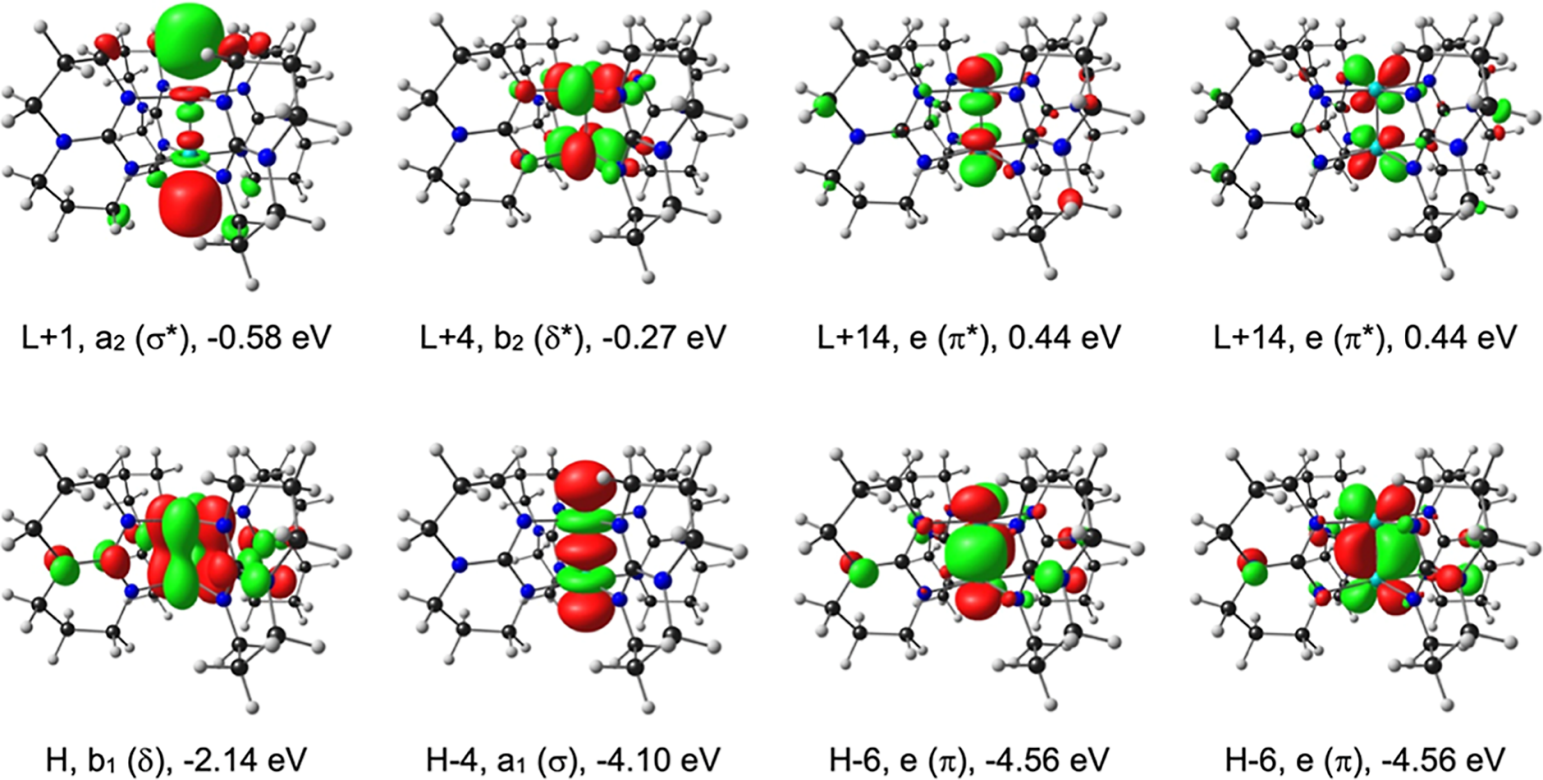
Selected OLYP-D3/ZORA-STO-TZ2P frontier MOs for Mo_2_(Hpp)_2_. H and L refer to HOMO and LUMO, respectively. Also shown are *D*_4_ irreps and Kohn–Sham orbital energies (eV).

Table 1 presents DFT-based IPs and electron affinities calculated with the ΔSCF method using different exchange–correlation functionals, namely, the classic pure functional BP86;^26,27^ the pure functional OLYP,^28,29^ which has often yielded improved results; and the hybrid functionals B3LYP^30^ and B3LYP*,^31,32^ with 20 and 15% Hartree–Fock exchange, respectively, all augmented with Grimme’s D3 dispersion corrections.^33^ Also listed in Table 1 are relevant experimental IPs, derived largely from gas-phase PES. Figure 1 presents a comparative MO energy level diagram for a selection of the compounds studied, namely, the two Hpp complexes and, for comparison, the two analogous porphyrin complexes.^18^Figure 2 depicts key metal-based OLYP-D3 frontier MOs for Mo_2_(Hpp)_4_ (the analogous MOs for the W_2_ complex are visually exceedingly similar and, accordingly, not shown). The results lead to the following conclusions.

**Table 1:** Calculated and Experimental IPs (eV) for the Molecules Studied^a^^,^^b^

	BP86-D3		OLYP-D3		B3LYP*-D3		B3LYP-D3		PES
	IP_v_	IP_a_	IP_v_	IP_a_	IP_v_	IP_a_	IP_v_	IP_a_	
Mo_2_(OFm)_4_ (*D*_4*h*_)	7.44	7.38	7.19	7.12	7.23	7.16	7.21	7.12	7.5^c^
W_2_(OFm)_4_ (*D*_4*h*_)	6.93	6.91	6.59	6.56	6.64	6.62	6.58	6.55	
Mo_2_(Me_2_Fa)_4_ (*D*_4*h*_)	5.36	5.30	5.10	5.04	5.11	5.04	5.08	4.99	5.63^d^
W_2_(Me_2_Fa)_4_ (*D*_4*h*_)	5.00	4.95	4.71	4.65	4.71	4.65	4.65	4.59	5.23^d^
Mo_2_(Hpp)_4_ (*D*_4_)	3.82	3.71	3.61	3.49	3.70	3.56	3.69	3.53	4.33 (4.01)^e^
W_2_(Hpp)_4_ (*D*_4_)	3.41	3.31	3.13	3.03	3.19	3.08	3.23	3.11	3.76 (3.51)^e^
{Mo[Por]}_2_ (*D*_4*h*_)	5.72	5.67	5.39	5.38	5.39		5.33	5.23	
{W[Por]}_2_ (*D*_4*h*_)	5.21		4.83	4.82	4.85		4.78		

a The calculations were carried out with a scalar-relativistic ZORA (zeroth order regular approximation to the Dirac equation)^34^ Hamiltonian, all-electron ZORA STO-TZ2P basis sets, fine integration grids and tight criteria for SCF and geometry optimization cycles, and appropriate point group symmetry, all as implemented in the ADF program system.^35^

b The subscripts v and a indicate “vertical” and “adiabatic”, respectively.

c Ref (7).

d Experimental measurements were carried out on *N*,*N*′-diphenylformamidinato (Ph_2_Fa) complexes; ref (13).

e The values within parentheses are the observed onset potentials; ref (14).

The present scalar-relativistic calculations with large Slater-type basis sets present some of the first *quantitative* insights (relative to early theoretical studies^10,13−15^) into the performance of DFT methods with respect to photoelectron spectra of classic metal–metal quadruple-bonded systems. Although we have long known that DFT methods do an impressive job of reproducing gas-phase IPs and electron affinities of organic and main-group systems (see selected studies from our laboratory^36−43^), the performance of DFT vis-à-vis transition-metal systems has been rather an open question. On the one hand, DFT methods have long struggled with reproducing the spin-state energetics of transition-metal complexes.^44−52^ On the other hand, DFT has an excellent track record of correctly predicting the redox site in metalloporphyrin-type complexes, such as nickel hydroporphyrins^53^ and a number of metal–metal multiple-bonded metallocorrole dimers.^54^ To our satisfaction, for Mo_2_(OFm)_4_, all four exchange–correlation functionals yielded vertical IPs in semiquantitative agreement with gas-phase PES, with the best agreement observed for BP86-D3. On the other hand, the calculated vertical IPs of the Hpp complexes are lower than the corresponding experimental values by ∼0.5 eV; interestingly, the errors relative to experimental “onset potentials” are much lower, only about 0.1–0.2 eV. We view these as rather modest errors that we can easily “live with”. More importantly, the calculations reproduce differences in IPs within pairs of analogous Mo_2_ and W_2_ complexes with near-quantitative accuracy. Overall, the four functionals examined appear to perform comparably, with the classic pure functional BP86 exhibiting the best agreement with gas-phase PES.

Experimentally, the first IPs span a > 4 eV range for dimolybdenum(II,II) paddlewheel complexes, from 4.33 eV for Mo_2_(Hpp)_4_14 to 8.76 eV for Mo_2_(CF_3_COO)_4_.11 For the analogous ditungsten(II,II) complexes, the IPs span a slightly smaller range of 3.63 eV, from 3.76 eV for W_2_(Hpp)_4_14 to 7.39 eV for W_2_(CF_3_COO)_4_.^11^ The calculations, regardless of the functional, appear to do an excellent job of reproducing the large ligand field effects on the experimentally observed IPs. The reason underlying the large ligand field effects seems rather obvious: in each case, the HOMO corresponds to the δ bond (see Figures 1 and 2), which is exclusively localized on the bimetal unit and, accordingly, highly susceptible to the ligands’ electronic effects.

Both calculated and experimental data reveal systematic differences between the IPs of analogous Mo_2_ and W_2_ complexes, with the vertical first IPs of the latter being lower by a margin of ∼0.5 eV (Table 1). Likewise, both Kohn–Sham orbital energy spectra and experimental PES measurements indicate that the same holds for metal–metal π-bonds.^13−15^ Based on comparisons between scalar-relativistic and nonrelativistic calculations with the same basis sets (as described in detail in earlier studies from our laboratory^21−24^), the differences in IPs between analogous Mo_2_ and W_2_ systems could be largely attributed to differences in relativistic effects for the two metals, with the W 5d orbitals significantly more destabilized by relativity than the Mo 4d orbitals. An interesting point is that the relativistic effects observed here are larger than, indeed almost twice, what we have observed for other analogous pairs of 4d and 5d element complexes.^21,24,25^ A plausible explanation appears to be that our earlier studies involved mononuclear complexes, whereas here we are concerned with a bimetal unit with the MOs in question derived from overlapping d orbitals from *two* metal atoms.

In contrast to the above, Figure 1 shows that metal–metal σ and σ* orbitals exhibit slightly *lower* orbital energies in W_2_ complexes than those in their Mo_2_ counterparts. This stabilization reflects the significant admixture of metal s character in these orbitals and the fact that the W 6s orbital is significantly more relativistically stabilized than the Mo 5s orbital.^19,20^ The relativistic destabilization of the δ* HOMO and the stabilization of the σ* LUMO/LUMO+1 in the W_2_ complexes relative to their Mo_2_ counterparts translate to significantly smaller HOMO–LUMO gaps for the former (Figure 1). Interestingly, as noted earlier,^18^ the LUMOs of the porphyrin complexes consist of a degenerate pair of porphyrin-based orbitals, which results in both exceedingly low HOMO–LUMO gaps and large electron affinities relative to the nonporphyrin complexes. In fact, according to our calculations, a positive EA is not predicted for any of the nonporphyrin-supporting ligands, except for small values < 0.5 eV for carboxylate-supporting ligands.

The scalar-relativistic calculations presented here predict different triplet states for Mo_2_ and W_2_ paddlewheel complexes. Taking the Hpp complexes as our paradigm, B3LYP*-D3 calculations on Mo_2_(Hpp)_4_ predict a δ^1^δ*^1^ triplet state at 1.09 eV and a δ^1^σ*^1^ state at 1.49 eV above the ground singlet state (both values refer to adiabatic energies). For W_2_(Hpp)_4_, in contrast, our calculations predict a lower-energy δ^1^σ*^1^ triplet state at 0.89 eV and a higher-energy δ^1^δ*^1^ triplet state at 1.45 eV, an interesting example of a relativity-driven reversal of excited-state energetics (see refs (5 and 6) for a general background).

## Conclusions

In summary, revisiting the gas-phase photoelectron spectra of quadruple-bonded dimolybdenum(II,II) and ditungsten(II,II) complexes with modern DFT methods has yielded a valuable calibration of four popular exchange–correlation functionals. In spite of a possible systematic error of a few tenths of an eV in the absolute values of the IPs, the functionals examined reproduce differences in IPs between analogous Mo_2_ and W_2_ complexes and large ligand field effects with near-quantitative accuracy. The calculations help us interpret a number of electronic differences between analogous Mo_2_ and W_2_ complexes in terms of differential relativistic effects. Thus, relativity results in not only lower IPs for the W_2_ complexes but also smaller HOMO–LUMO gaps and different triplet states relative to their Mo_2_ counterparts.

## Supporting Information Available

Optimized DFT coordinates (PDF)
