# Supplementary material for: Theoretical Photoelectron Spectroscopy of Quadruple-Bonded Dimolybdenum(II,II) and Ditungsten(II,II) Paddlewheel Complexes: Performance of Common Density Functional Theory Methods
Source: ACS Omega. 2024 Mar 4;9(10):12237–41. doi: 10.1021/acsomega.4c00269 (PMC10938323; doi:10.1021/acsomega.4c00269)
Supplement: Supplementary file 1 — ao4c00269_si_001.pdf [file ao4c00269_si_001.pdf]

Supporting information

Theoretical Photoelectron Spectroscopy of Quadruple-Bonded Dimolybdenum(II,II) and Ditungsten(II,II) Paddlewheel Complexes: Performance of Common Density Functional Theory Methods

Abhik Ghosh*[,a] and Jeanet Conradie*[,a,b]

[a] Department of Chemistry, UiT – The Arctic University of Norway, N-9037 Tromsø, Norway

[b] Department of Chemistry, University of the Free State, P.O. Box 339, Bloemfontein 9300, Republic of South Africa

All-electron B3LYP-D3/ZORA-STO-TZ2P optimized Cartesian coordinates (Å)

Table of Contents

1. Mo_2HPP_4, D_4, q = 0, S = 0 2
2. W_2HPP_4, D_4, q = 0, S = 0 3
3. $Mo_2(OFm)_4$, D_{4h}, q = 0, S = 0 5
4. $W_2(OFm)_4$, D_{4h}, q = 0, S = 0 6
5. $Mo_2(Me_2Fa)_4$, D_{4h}, q = 0, S = 0 6
6. $W_2(Me_2Fa)_4$, D_{4h}, q = 0, S = 0 7

1. Mo_2HPP_4, D_4, q = 0, S = 0

Mo	0.000000000	0.000000000	1.028574000
Mo	0.000000000	0.000000000	-1.028574000
C	1.962611000	2.147599000	-2.387314000
C	1.962611000	-2.147599000	2.387314000
C	2.020389000	2.020389000	0.000000000
C	2.020389000	-2.020389000	0.000000000
C	2.147599000	1.962611000	2.387314000
C	2.147599000	-1.962611000	-2.387314000
C	2.429869000	3.460797000	2.349509000
C	2.429869000	-3.460797000	-2.349509000
C	3.383190000	3.738994000	1.197781000
C	3.383190000	-3.738994000	-1.197781000
C	3.460797000	2.429869000	-2.349509000
C	3.460797000	-2.429869000	2.349509000
C	3.738994000	3.383190000	-1.197781000
C	3.738994000	-3.383190000	1.197781000
C	-1.962611000	2.147599000	2.387314000
C	-1.962611000	-2.147599000	-2.387314000
C	-2.020389000	2.020389000	0.000000000
C	-2.020389000	-2.020389000	0.000000000
C	-2.147599000	1.962611000	-2.387314000
C	-2.147599000	-1.962611000	2.387314000
C	-2.429869000	3.460797000	-2.349509000
C	-2.429869000	-3.460797000	2.349509000
C	-3.383190000	3.738994000	-1.197781000
C	-3.383190000	-3.738994000	1.197781000
C	-3.460797000	2.429869000	2.349509000
C	-3.460797000	-2.429869000	-2.349509000
C	-3.738994000	3.383190000	1.197781000
C	-3.738994000	-3.383190000	-1.197781000
H	1.420194000	3.087032000	-2.589527000
H	1.420194000	-3.087032000	2.589527000
H	1.469075000	1.725301000	3.212043000
H	1.469075000	-1.725301000	-3.212043000
H	1.485786000	3.991588000	2.196119000
H	1.485786000	-3.991588000	-2.196119000
H	1.725301000	1.469075000	-3.212043000
H	1.725301000	-1.469075000	3.212043000
H	2.870199000	3.814061000	3.285656000
H	2.870199000	-3.814061000	-3.285656000
H	3.087032000	1.420194000	2.589527000
H	3.087032000	-1.420194000	-2.589527000
H	3.402174000	4.812746000	0.964262000
H	3.402174000	-4.812746000	-0.964262000
H	3.464600000	4.409239000	-1.489799000
H	3.464600000	-4.409239000	1.489799000
H	3.814061000	2.870199000	-3.285656000
H	3.814061000	-2.870199000	3.285656000
H	3.991588000	1.485786000	-2.196119000

H	3.991588000	-1.485786000	2.196119000
H	4.409239000	3.464600000	1.489799000
H	4.409239000	-3.464600000	-1.489799000
H	4.812746000	3.402174000	-0.964262000
H	4.812746000	-3.402174000	0.964262000
H	-1.420194000	3.087032000	2.589527000
H	-1.420194000	-3.087032000	-2.589527000
H	-1.469075000	1.725301000	-3.212043000
H	-1.469075000	-1.725301000	3.212043000
H	-1.485786000	3.991588000	-2.196119000
H	-1.485786000	-3.991588000	2.196119000
H	-1.725301000	1.469075000	3.212043000
H	-1.725301000	-1.469075000	-3.212043000
H	-2.870199000	3.814061000	-3.285656000
H	-2.870199000	-3.814061000	3.285656000
H	-3.087032000	1.420194000	-2.589527000
H	-3.087032000	-1.420194000	2.589527000
H	-3.402174000	4.812746000	-0.964262000
H	-3.402174000	-4.812746000	0.964262000
H	-3.464600000	4.409239000	1.489799000
H	-3.464600000	-4.409239000	-1.489799000
H	-3.814061000	2.870199000	3.285656000
H	-3.814061000	-2.870199000	-3.285656000
H	-3.991588000	1.485786000	2.196119000
H	-3.991588000	-1.485786000	-2.196119000
H	-4.409239000	3.464600000	-1.489799000
H	-4.409239000	-3.464600000	1.489799000
H	-4.812746000	3.402174000	0.964262000
H	-4.812746000	-3.402174000	-0.964262000
N	1.522163000	1.542823000	-1.145272000
N	1.522163000	-1.542823000	1.145272000
N	1.542823000	1.522163000	1.145272000
N	1.542823000	-1.522163000	-1.145272000
N	3.006206000	3.006206000	0.000000000
N	3.006206000	-3.006206000	0.000000000
N	-1.522163000	1.542823000	1.145272000
N	-1.522163000	-1.542823000	-1.145272000
N	-1.542823000	1.522163000	-1.145272000
N	-1.542823000	-1.522163000	1.145272000
N	-3.006206000	3.006206000	0.000000000
N	-3.006206000	-3.006206000	0.000000000

2. W_2HPP_4, D_4, q = 0, S = 0

W	0.000000000	0.000000000	1.079351000
W	0.000000000	0.000000000	-1.079351000
C	1.975600000	2.124040000	-2.387806000
C	1.975600000	-2.124040000	2.387806000
C	2.003575000	2.003575000	0.000000000

C	2.003575000	-2.003575000	0.000000000
C	2.124040000	1.975600000	2.387806000
C	2.124040000	-1.975600000	-2.387806000
C	2.368959000	3.479333000	2.329832000
C	2.368959000	-3.479333000	-2.329832000
C	3.329088000	3.760895000	1.184234000
C	3.329088000	-3.760895000	-1.184234000
C	3.479333000	2.368959000	-2.329832000
C	3.479333000	-2.368959000	2.329832000
C	3.760895000	3.329088000	-1.184234000
C	3.760895000	-3.329088000	1.184234000
C	-1.975600000	2.124040000	2.387806000
C	-1.975600000	-2.124040000	-2.387806000
C	-2.003575000	2.003575000	0.000000000
C	-2.003575000	-2.003575000	0.000000000
C	-2.124040000	1.975600000	-2.387806000
C	-2.124040000	-1.975600000	2.387806000
C	-2.368959000	3.479333000	-2.329832000
C	-2.368959000	-3.479333000	2.329832000
C	-3.329088000	3.760895000	-1.184234000
C	-3.329088000	-3.760895000	1.184234000
C	-3.479333000	2.368959000	2.329832000
C	-3.479333000	-2.368959000	-2.329832000
C	-3.760895000	3.329088000	1.184234000
C	-3.760895000	-3.329088000	-1.184234000
H	1.413293000	3.982571000	2.157567000
H	1.413293000	-3.982571000	-2.157567000
H	1.452436000	1.730195000	3.215654000
H	1.452436000	-1.730195000	-3.215654000
H	1.457485000	3.076148000	-2.590225000
H	1.457485000	-3.076148000	2.590225000
H	1.730195000	1.452436000	-3.215654000
H	1.730195000	-1.452436000	3.215654000
H	2.789707000	3.858845000	3.264629000
H	2.789707000	-3.858845000	-3.264629000
H	3.076148000	1.457485000	2.590225000
H	3.076148000	-1.457485000	-2.590225000
H	3.322635000	4.830234000	0.928410000
H	3.322635000	-4.830234000	-0.928410000
H	3.521130000	4.358116000	-1.496219000
H	3.521130000	-4.358116000	1.496219000
H	3.858845000	2.789707000	-3.264629000
H	3.858845000	-2.789707000	3.264629000
H	3.982571000	1.413293000	-2.157567000
H	3.982571000	-1.413293000	2.157567000
H	4.358116000	3.521130000	1.496219000
H	4.358116000	-3.521130000	-1.496219000
H	4.830234000	3.322635000	-0.928410000
H	4.830234000	-3.322635000	0.928410000
H	-1.413293000	3.982571000	-2.157567000
H	-1.413293000	-3.982571000	2.157567000

H	-1.452436000	1.730195000	-3.215654000
H	-1.452436000	-1.730195000	3.215654000
H	-1.457485000	3.076148000	2.590225000
H	-1.457485000	-3.076148000	-2.590225000
H	-1.730195000	1.452436000	3.215654000
H	-1.730195000	-1.452436000	-3.215654000
H	-2.789707000	3.858845000	-3.264629000
H	-2.789707000	-3.858845000	3.264629000
H	-3.076148000	1.457485000	-2.590225000
H	-3.076148000	-1.457485000	2.590225000
H	-3.322635000	4.830234000	-0.928410000
H	-3.322635000	-4.830234000	0.928410000
H	-3.521130000	4.358116000	1.496219000
H	-3.521130000	-4.358116000	-1.496219000
H	-3.858845000	2.789707000	3.264629000
H	-3.858845000	-2.789707000	-3.264629000
H	-3.982571000	1.413293000	2.157567000
H	-3.982571000	-1.413293000	-2.157567000
H	-4.358116000	3.521130000	-1.496219000
H	-4.358116000	-3.521130000	1.496219000
H	-4.830234000	3.322635000	0.928410000
H	-4.830234000	-3.322635000	-0.928410000
N	1.508107000	1.523976000	-1.150242000
N	1.508107000	-1.523976000	1.150242000
N	1.523976000	1.508107000	1.150242000
N	1.523976000	-1.508107000	-1.150242000
N	2.990423000	2.990423000	0.000000000
N	2.990423000	-2.990423000	0.000000000
N	-1.508107000	1.523976000	1.150242000
N	-1.508107000	-1.523976000	-1.150242000
N	-1.523976000	1.508107000	-1.150242000
N	-1.523976000	-1.508107000	1.150242000
N	-2.990423000	2.990423000	0.000000000
N	-2.990423000	-2.990423000	0.000000000

3. $Mo_2(OFm)_4$, D_{4h}, q = 0, S = 0

Mo	0.000000000	0.000000000	1.038656000
Mo	0.000000000	0.000000000	-1.038656000
C	0.000000000	2.707298000	0.000000000
C	0.000000000	-2.707298000	0.000000000
C	2.707298000	0.000000000	0.000000000
C	-2.707298000	0.000000000	0.000000000
H	0.000000000	3.801420000	0.000000000
H	0.000000000	-3.801420000	0.000000000
H	3.801420000	0.000000000	0.000000000
H	-3.801420000	0.000000000	0.000000000
O	0.000000000	2.106576000	1.116268000

O	0.000000000	2.106576000	-1.116268000
O	0.000000000	-2.106576000	1.116268000
O	0.000000000	-2.106576000	-1.116268000
O	2.106576000	0.000000000	1.116268000
O	2.106576000	0.000000000	-1.116268000
O	-2.106576000	0.000000000	1.116268000
O	-2.106576000	0.000000000	-1.116268000

4. $W_2(OFm)_4$, D_{4h}, q = 0, S = 0

W	0.000000000	0.000000000	1.086844000
W	0.000000000	0.000000000	-1.086844000
C	0.000000000	2.689861000	0.000000000
C	0.000000000	-2.689861000	0.000000000
C	2.689861000	0.000000000	0.000000000
C	-2.689861000	0.000000000	0.000000000
H	0.000000000	3.781306000	0.000000000
H	0.000000000	-3.781306000	0.000000000
H	3.781306000	0.000000000	0.000000000
H	-3.781306000	0.000000000	0.000000000
O	0.000000000	2.084780000	1.121660000
O	0.000000000	2.084780000	-1.121660000
O	0.000000000	-2.084780000	1.121660000
O	0.000000000	-2.084780000	-1.121660000
O	2.084780000	0.000000000	1.121660000
O	2.084780000	0.000000000	-1.121660000
O	-2.084780000	0.000000000	1.121660000
O	-2.084780000	0.000000000	-1.121660000

5. $Mo_2(Me_2Fa)_4$, D_{4h}, q = 0, S = 0

Mo	0.000000000	0.000000000	1.042050000
Mo	0.000000000	0.000000000	-1.042050000
C	1.981904000	1.981904000	0.000000000
C	1.981904000	-1.981904000	0.000000000
C	2.084586000	2.084586000	2.370953000
C	2.084586000	2.084586000	-2.370953000
C	2.084586000	-2.084586000	2.370953000
C	2.084586000	-2.084586000	-2.370953000
C	-1.981904000	1.981904000	0.000000000
C	-1.981904000	-1.981904000	0.000000000
C	-2.084586000	2.084586000	2.370953000
C	-2.084586000	2.084586000	-2.370953000
C	-2.084586000	-2.084586000	2.370953000
C	-2.084586000	-2.084586000	-2.370953000

H	1.617989000	1.617989000	3.240833000
H	1.617989000	1.617989000	-3.240833000
H	1.617989000	-1.617989000	3.240833000
H	1.617989000	-1.617989000	-3.240833000
H	1.908570000	3.164732000	2.449675000
H	1.908570000	3.164732000	-2.449675000
H	1.908570000	-3.164732000	2.449675000
H	1.908570000	-3.164732000	-2.449675000
H	2.758311000	2.758311000	0.000000000
H	2.758311000	-2.758311000	0.000000000
H	3.164732000	1.908570000	2.449675000
H	3.164732000	1.908570000	-2.449675000
H	3.164732000	-1.908570000	2.449675000
H	3.164732000	-1.908570000	-2.449675000
H	-1.617989000	1.617989000	3.240833000
H	-1.617989000	1.617989000	-3.240833000
H	-1.617989000	-1.617989000	3.240833000
H	-1.617989000	-1.617989000	-3.240833000
H	-1.908570000	3.164732000	2.449675000
H	-1.908570000	3.164732000	-2.449675000
H	-1.908570000	-3.164732000	2.449675000
H	-1.908570000	-3.164732000	-2.449675000
H	-2.758311000	2.758311000	0.000000000
H	-2.758311000	-2.758311000	0.000000000
H	-3.164732000	1.908570000	2.449675000
H	-3.164732000	1.908570000	-2.449675000
H	-3.164732000	-1.908570000	2.449675000
H	-3.164732000	-1.908570000	-2.449675000
N	1.522870000	1.522870000	1.151499000
N	1.522870000	1.522870000	-1.151499000
N	1.522870000	-1.522870000	1.151499000
N	1.522870000	-1.522870000	-1.151499000
N	-1.522870000	1.522870000	1.151499000
N	-1.522870000	1.522870000	-1.151499000
N	-1.522870000	-1.522870000	1.151499000
N	-1.522870000	-1.522870000	-1.151499000

6. $W_2(Me_2Fa)_4$, D_{4h}, q = 0, S = 0

W	0.000000000	0.000000000	1.091266000
W	0.000000000	0.000000000	-1.091266000
C	1.969703000	1.969703000	0.000000000
C	1.969703000	-1.969703000	0.000000000
C	2.077301000	2.077301000	2.373738000
C	2.077301000	2.077301000	-2.373738000
C	2.077301000	-2.077301000	2.373738000
C	2.077301000	-2.077301000	-2.373738000
C	-1.969703000	1.969703000	0.000000000
C	-1.969703000	-1.969703000	0.000000000

C	-2.077301000	2.077301000	2.373738000
C	-2.077301000	2.077301000	-2.373738000
C	-2.077301000	-2.077301000	2.373738000
C	-2.077301000	-2.077301000	-2.373738000
H	1.613138000	1.613138000	3.246043000
H	1.613138000	1.613138000	-3.246043000
H	1.613138000	-1.613138000	3.246043000
H	1.613138000	-1.613138000	-3.246043000
H	1.900719000	3.157096000	2.447384000
H	1.900719000	3.157096000	-2.447384000
H	1.900719000	-3.157096000	2.447384000
H	1.900719000	-3.157096000	-2.447384000
H	2.744273000	2.744273000	0.000000000
H	2.744273000	-2.744273000	0.000000000
H	3.157096000	1.900719000	2.447384000
H	3.157096000	1.900719000	-2.447384000
H	3.157096000	-1.900719000	2.447384000
H	3.157096000	-1.900719000	-2.447384000
H	-1.613138000	1.613138000	3.246043000
H	-1.613138000	1.613138000	-3.246043000
H	-1.613138000	-1.613138000	3.246043000
H	-1.613138000	-1.613138000	-3.246043000
H	-1.900719000	3.157096000	2.447384000
H	-1.900719000	3.157096000	-2.447384000
H	-1.900719000	-3.157096000	2.447384000
H	-1.900719000	-3.157096000	-2.447384000
H	-2.744273000	2.744273000	0.000000000
H	-2.744273000	-2.744273000	0.000000000
H	-3.157096000	1.900719000	2.447384000
H	-3.157096000	1.900719000	-2.447384000
H	-3.157096000	-1.900719000	2.447384000
H	-3.157096000	-1.900719000	-2.447384000
N	1.508935000	1.508935000	1.156804000
N	1.508935000	1.508935000	-1.156804000
N	1.508935000	-1.508935000	1.156804000
N	1.508935000	-1.508935000	-1.156804000
N	-1.508935000	1.508935000	1.156804000
N	-1.508935000	1.508935000	-1.156804000
N	-1.508935000	-1.508935000	1.156804000
N	-1.508935000	-1.508935000	-1.156804000
